# L-cystathionine protects against oxidative stress and DNA damage induced by oxidized low-density lipoprotein in THP-1-derived macrophages

**DOI:** 10.3389/fphar.2023.1161542

**Published:** 2023-07-25

**Authors:** Hanlin Peng, Mingzhu Zhu, Wei Kong, Chaoshu Tang, Junbao Du, Yaqian Huang, Hongfang Jin

**Affiliations:** ^1^ Department of Pediatrics, Peking University First Hospital, Beijing, China; ^2^ Department of Physiology and Pathophysiology, Peking University Health Science Center, Beijing, China; ^3^ State Key Laboratory of Vascular Homeostasis and Remodeling, Peking University, Beijing, China

**Keywords:** L-cystathionine, oxidative stress, macrophage, oxidized low-density lipoprotein, DNA damage

## Abstract

**Introduction:** Oxidative stress in monocyte-derived macrophages is a significant pathophysiological process in atherosclerosis. L-cystathionine (L-Cth) acts as a scavenger for oxygen free radicals. However, the impact of L-Cth on macrophage oxidative stress during atherogenesis has remained unclear. This study aimed to investigate whether L-Cth affects oxidative stress in THP-1-derived macrophages and its subsequent effects on DNA damage and cell apoptosis.

**Methods:** We established a cellular model of oxLDL-stimulated macrophages. The content of superoxide anion, H_2_O_2_, NO, and H_2_S in the macrophage were in situ detected by the specific fluorescence probe, respectively. The activities of SOD, GSH-Px, and CAT were measured by colorimetrical assay. The protein expressions of SOD1, SOD2, and iNOS were detected using western blotting. The DNA damage and apoptosis in the macrophage was evaluated using an fluorescence kit.

**Results:** The results demonstrated that oxLDL significantly increased the content of superoxide anion and H_2_O_2_, the expression of iNOS protein, and NO production in macrophages. Conversely, oxLDL decreased the activity of antioxidants GSH-Px, SOD, and CAT, and downregulated the protein expressions of SOD1 and SOD2 in macrophages. However, treatment with L-Cth reduced the levels of superoxide anion, H_2_O_2_, and NO, as well as the protein expression of iNOS induced by oxLDL. Moreover, L-Cth treatment significantly enhanced GSH-Px, SOD, and CAT activity, and upregulated the expressions of SOD1 and SOD2 proteins in macrophages treated with oxLDL. Furthermore, both L-Cth supplementation and activation of endogenous L-Cth production suppressed DNA damage and cell apoptosis in oxLDL-injured macrophages, whereas inhibition of endogenous L-Cth exacerbated the deleterious effects of oxLDL.

**Conclusion:** These findings suggest that L-Cth exerts a pronounced inhibitory effect on the oxidative stress, subsequent DNA damage and cell apoptosis in oxLDL-stimulated THP-1 monocytes. This study deepens our understanding of the pathogenesis of macrophage-related cardiovascular pathology.

## 1 Introduction

Atherosclerosis serves as the underlying pathology for numerous cardiovascular and cerebrovascular diseases. The incidence and mortality of associated cardiovascular events have increased significantly over the decades, imposing a substantial burden on individual health (Xu et al., 2019; Soehnlein and Libby, 2021; Bjorkegren and Lusis, 2022). Recent findings indicate that oxidative stress in monocyte-derived macrophages is the primary instigator of atherosclerotic damage and a key contributor to the pathogenesis of atherosclerosis (Cominacini et al., 2015; Yang et al., 2017). Although the regulatory mechanisms governing macrophage oxidative stress in atherogenesis have been extensively investigated, they have not yet been fully elucidated. During macrophage oxidative stress, oxygen free radicals can induce oxidative modifications in low-density lipoprotein (LDL). Recognizing and internalizing oxidized LDL (oxLDL) by macrophage scavenger receptors led to the formation of foam cells derived from macrophages. These foam cells continue to accumulate oxLDL until they undergo necrosis and disintegration, releasing LDL outside the cells and forming a lipid core that contributes to atherosclerotic damage (Poznyak et al., 2020a; Khatana et al., 2020). Moreover, oxidative stress can cause DNA damage in macrophages, activate p53, induce growth arrest and apoptosis in macrophages, promote necrotic core formation, and ultimately lead to plaque rupture (Bennett, 2001). Consequently, the quest for endogenous intervention strategies to counteract macrophage oxidative damage has gained significant attention worldwide.

Studies have demonstrated that various intermediate and end metabolites generated during sulfur-containing amino acid metabolism form a distinct family of sulfur-containing amino acids, which is widely involved in the development of oxidative stress in the atherosclerotic vessels. For instance, homocysteine can impair vascular endothelial cells, stimulate clonal proliferation of lymphocytes, and provoke and exacerbate vascular oxidative stress through various mechanisms (Zhang et al., 2002). L-cystathionine (L-Cth), (2*S*)-2-amino-4-[(2*R*)-2-amino-2-carboxyethyl] sulfanylbutanoic acid, is an amino acid synthesized through the conversion of methionine to cysteine *in vivo* (Gaull et al., 1972). L-Cth serves as an important intermediate in the metabolism of sulfur-containing amino acids, although limited research has explored its relatively independent biological effects. Reports indicate that L-Cth not only dose-dependently reduces the superoxide anion (O_2_^−^) produced by human leukocytes *in vitro* (ranging from 30 μM to 10 mM), but also directly scavenges O_2_^−^ derived from the xanthine-xanthine oxidase system (Wada et al., 1996). Furthermore, intraperitoneal injection of L-Cth inhibits gastric mucosal erosion and lipid peroxidation in ischemia-reperfusion rats, with a more pronounced effect than other free radical scavengers like cysteine and superoxide dismutase (SOD) (Wada et al., 1995). These studies suggest that L-Cth may act as an oxygen free radical scavenger. However, the precise mechanism by which L-Cth regulates macrophage oxidative stress during atherogenesis has not yet been unclear.

The goal of the present study was to investigate how L-Cth affects oxidative stress and subsequent DNA damage induced by oxLDL in THP-1 monocyte-derived macrophages, with the aim of understanding the mechanisms by which L-Cth modulates the oxidative stress response of macrophages and its cellular protective roles.

## 2 Materials and methods

### 2.1 Cell culture

Human monocyte derived from patients with acute monocytic leukemia, specifically THP-1 cell (Procell, Wuhan, China), was used in the study. The chemicals utilized in this study, along with the corresponding companies from which they were purchased, are listed in Supplementary Table S1. The differentiation from THP-1 cell to adherent macrophage was induced by an incubation with 50 nM phorbol 12-myristate 13-acetate (PMA, REF: P8139; Sigma, United States) for 24 h. To synchronize the cells, the differentiated macrophages were cultured in basal RPMI 1640 medium for 24 h and then allocated into the following groups: control, oxLDL, oxLDL + L-Cth 0.1 mM, oxLDL + L-Cth 0.3 mM, and oxLDL + L-Cth 1.0 mM. OxLDL and L-Cth were procured from Zhongshan Golden Bridge and Sigma-Aldrich, respectively. In the oxLDL group, 50 mg/L oxLDL was added and incubated for 6 h. In the oxLDL + L-Cth 0.1 mM group, oxLDL + L-Cth 0.3 mM group, and oxLDL + L-Cth 1.0 mM group, the cells were pre-treated with L-Cth for 30 min and subsequently exposed to 50 mg/L oxLDL for 6 h (Zhu et al., 2014). Likewise, in the oxLDL + aminooxy acetic acid (AOAA; REF: S4989; Selleck, United States) group and the oxLDL + S-adenosylmethionine (SAM; REF: S910367; Macklin, China) group, the cells were intervened with AOAA or SAM for 30 min, followed by a treatment with 50 mg/L oxLDL for 6 h.

### 2.2 DNA damage detection

The phosphorylated H2A histone family member X (γ-H2AX) immunofluorescence kit (REF: C2035S; Beyotime, Shanghai, China) was utilized to assess cellular DNA damage (Li et al., 2022). The culture medium was aspirated, and fixing solution was added for a duration of 15 min. After rinsing with detergent three times for 10 min each, an immunostaining blocking solution was applied and incubated for 20 min. Subsequently, the first antibody for γ-H2AX was incubated overnight at 4°C following the removal of the immunostaining blocking solution. Following three washes with detergent, the second antibody combined with Alexa-fluor488 was incubated at room temperature for 1 h. After the cells were rinsed twice, DAPI was added and incubated for 5 min. The green fluorescence signals were observed with an excitation wavelength of 488 nm and an emission wavelength of 519 nm under a laser confocal microscope (objective: ×63; Leica, Germany).

### 2.3 Detection of O_2_^−^ content using dihydroethidium fluorescence probe

At the end of the cell experiments, the cells were washed with phosphate-buffered saline (PBS). Subsequently, a 10 µM dihydroethidium (DHE) probe (REF: S0063; Beyotime, Shanghai, China) was added and incubated at 37°C for 30 min in a dark cabin (Wei et al., 2010). A confocal laser scanning microscope (objective: ×40; Leica, Germany) was used to visualize the red fluorescence emitted by the probe, with an excitation wavelength of 535 nm.

### 2.4 Detection of H_2_O_2_ content using BES-H_2_O_2_-Ac fluorescence probe

The H_2_O_2_-specific fluorescent probe BES-H_2_O_2_-Ac (REF: 028-17811; Wako, Osaka, Japan) was dissolved in dimethyl sulfoxide (Kanzaki et al., 2014). Briefly, after an incubation with 5 µM BES-H_2_O_2_-Ac probe (working concentration) for 30 min in a 37°C incubator, the remaining free probe was removed by rinsing with PBS. The H_2_O_2_-specific fluorescence was observed under a confocal laser scanning microscope (objective: ×40; Leica, Germany) by setting excitation and emission wavelength as 485 nm and 515 nm, respectively.

### 2.5 Detection of NO content using DAF-FM DA fluorescence probe

The nitric oxide (NO) quantitative detection fluorescence probe DAF-FM DA (REF: S0019; Beyotime, Shanghai, China) was used to detect the NO content in cells at a working concentration of 10 µM (Chen et al., 2019). The culture medium was aspirated, and the diluted probe was added. Cells were rinsed with PBS after the incubation for 20 min in a 37°C incubator to ensure sufficient removal of DAF-FM DA. The observation was conducted under a confocal laser scanning microscope (objective: ×40; Leica, Germany) using an excitation and emission wavelength as 495 nm and 515 nm, repsectively.

### 2.6 Determination of glutathione peroxidase (GSH-Px), superoxide dismutase (SOD), and catalase (CAT) activities in the cell

The activities of GSH-Px, SOD, and CAT were determined by the colorimetric method. The GSH-Px assay kit (REF: A005-1-2; Nanjing Jiancheng, Nanjing, China), SOD assay kit (REF: BC0175; Solarbio, Beijing, China), and CAT assay kit (REF: A007-1-1; Nanjing Jiancheng, Nanjing, China) were used (Zhang M. et al., 2014; Zhang JQ. et al., 2014; Peng et al., 2022). Briefly, the cells were rinsed with PBS and subsequently lysed on ice for 10 min. Total protein was collected by a centrifugation at 12,000 g and 4°C. The protein concentration was measured for normalizing the activity.

The principle of detecting GSH-Px activity is based on a GSH-Px-catalyzed reaction of reduced glutathione (GSH) with hydrogen peroxide (H_2_O_2_) to generate water and oxidized glutathione (GSSG). The substrate GSH can react with Ellman’s reagent and then produce 2-Nitro-5-thiobenzoate anion with a high absorbance coefficient at 412 nm in a microplate reader (Varioskan LUX, Thermo Scientific, United States). By measuring the reduction in GSH levels, the GSH-Px activity in the sample was calculated.

SOD can scavenge the O_2_^−^ produced by the xanthine oxidative coupling reaction system. O_2_^−^ reduces nitro blue tetrazolium to formazan which exhibits a high absorbance coefficient at 560 nm. Therefore, SOD activity was determined by a decrease in the absorbance in a microplate reader (Varioskan LUX, Thermo Scientific, United States).

The decomposition of H_2_O_2_ by CAT is rapidly halted by ammonium molybdate. Moreover, ammonium molybdate can react with the leftover H_2_O_2_ to form a yellow end-product which can be quantified by measuring the absorbance at 405 nm (Varioskan LUX, Thermo Scientific, United States). Thus, the CAT activity was calculated based on the decrease in absorbance at 405 nm.

### 2.7 Determination of SOD1, SOD2, and iNOS protein expressions by Western blotting

The protein expressions of SOD1, SOD2, and inducible nitric oxide synthase (iNOS) in THP-1 monocyte-derived human macrophages were quantitatively analyzed in the following experimental groups: control group, oxLDL group, oxLDL + L-Cth 0.1 mM group, oxLDL + L-Cth 0.3 mM group, and oxLDL + L-Cth 1.0 mM group. Western blotting was performed according to previously described methods (Huang et al., 2016). The cells were treated and subsequently lysed in RIPA buffer (REF: P0013B; Beyotime, Shanghai, China). After a centrifugation at 12,000 g for 10 min at 4°C, the supernatant was collected. The total protein was mixed with loading buffer, separated through SDS-PAGE electrophoresis, and transferred on a nitrocellulose membrane (Amersham, United States). Skimmed milk was used for blocking at room temperature for 1 h. Subsequently, the corresponding primary antibodies were incubated with the nitrocellulose membrane at 4°C overnight, respectively. The primary antibodies were freshly diluted in PBST. The dilute proportions were listed as follows: iNOS (1:1000, REF: 13,120; CST, United States), SOD1 (1:2000, REF: ADI-SOD-100-D; Enzo, United States), SOD2 (1:2000, REF: ADI-SOD-200-D; Enzo, United States), and GAPDH (1:2000, REF: KC-5G4, Kangcheng, China). And then, the unbinding primary antibodies were removed by washing with PBST. The corresponding secondary antibodies were incubated at room temperature for 1 h with a dilute proportion of 1:5000. Finally, the protein band was visualized under a FluorChem M MultiFluor System (ProteinSimple, San Francisco, CA, United States) following a rapid incubation with ECL chemiluminescence reagent (REF: MA0186; MeilunBio, China).

### 2.8 Detection of H_2_S content using fluorescence probe

We employed the H_2_S-specific fluorescent probe SF7-AM (REF:14623, Cayman, Ann Arbor, United States) to measure the cellular H_2_S content (Lin et al., 2013). The cells were washed with PBS. Subsequently, a solution of 10 μM H_2_S fluorescent probe was incubated with the cells in an incubator at 37°C for 30 min. Finally, a 15-min fixation at room temperature was conducted using 4% paraformaldehyde. Nuclei staining was performed using DAPI dye. The confocal laser scanning microscope (objective: ×63; Leica, Germany) was utilized to capture the green and blue fluorescence.

### 2.9 *In situ* detection of apoptosis by terminal deoxynucleotidyl transferase dUTP nick-end labeling (TUNEL) assay

A commercial TUNEL kit (REF: 12156792910, Roche, Basel, Switzerland) was used to detect the cell apoptosis as previously reported (Du et al., 2018). Briefly, the cells were fixed with 4% paraformaldehyde for 15 min. Subsequently, they were covered by a permeabilization solution (0.25 g of BSA, 15 μL of Triton X-100, and 5 mL of PBS) at 37°C for 30 min. And then, the cells were incubated with the TUNEL reaction mixture in the dark for 1 h at 37°C. Finally, the cellular nuclei were counterstained with DAPI dye. The TUNEL-positive cells were observed under a confocal laser scanning microscope (objective: ×40; Leica, Germany), with an excitation and emission wavelength setting at 450–500 nm and 515–565 nm, respectively.

### 2.10 Statistics

SPSS software (version 22.0) was used for data analysis. The results were presented as mean ± standard deviation. One-way ANOVA was employed to compare the differences among multiple groups and LSD testing was subsequently conducted for further comparisons. The statistically significance was defined when *p*-value was less than 0.05.

## 3 Results

### 3.1 L-Cth inhibited oxLDL-stimulated O_2_^−^ and H_2_O_2_ production in THP-1 monocyte-derived human macrophages

**FIGURE 1:**
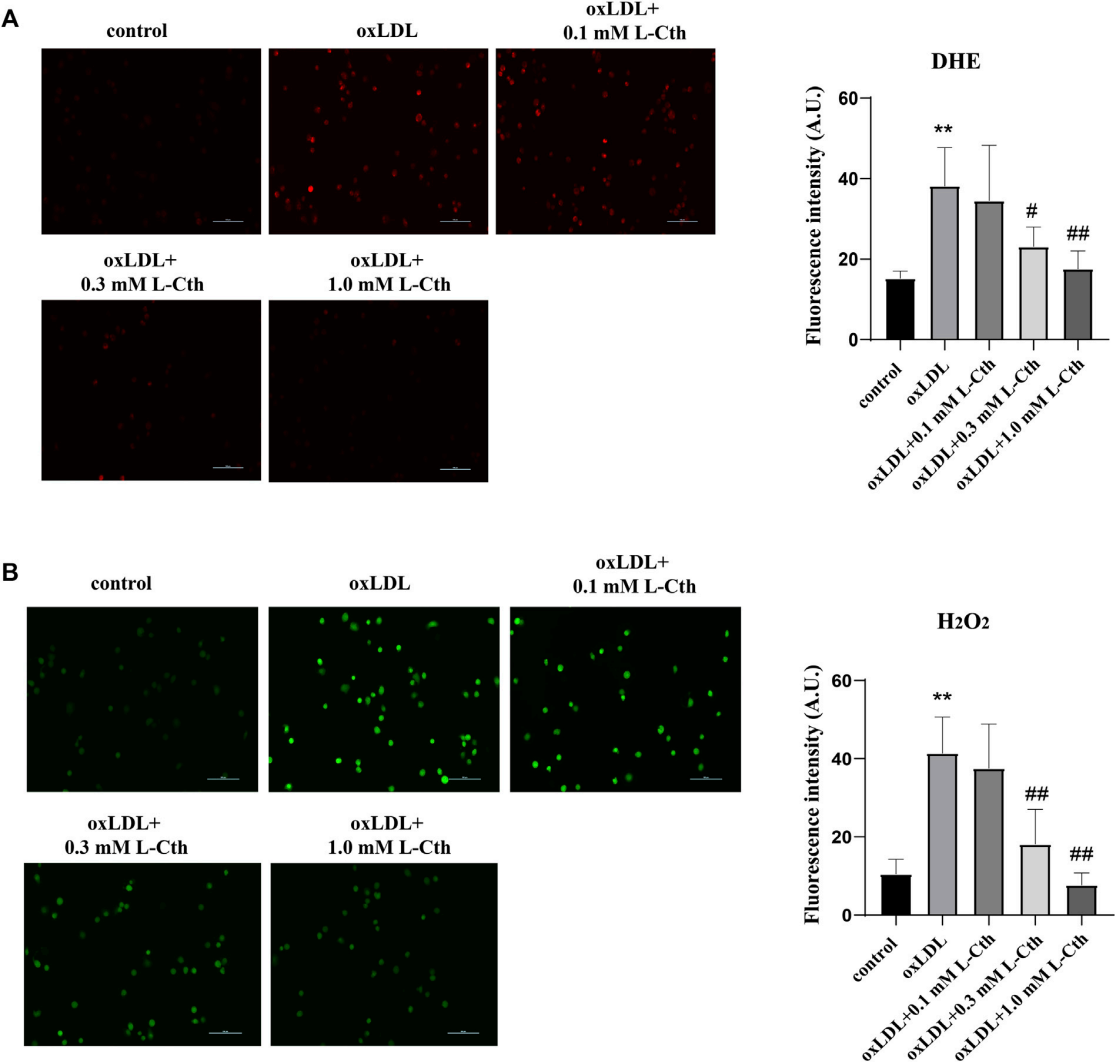
L-Cth inhibited oxLDL-stimulated production of superoxide anion and H_2_O_2_ in THP-1 monocyte-derived human macrophages. **(A)** The superoxide anion fluorescence probe (DHE) was used to identify superoxide anion levels in each group. **(B)** The fluorescence probe was used to detect H_2_O_2_ levels in each group. These data were from three independent cultures. **p < 0.01, vs. control group; ^##^ *p* < 0.01, ^#^ *p* < 0.05, vs. oxLDL group.

To investigate the regulatory effect of L-Cth on oxygen free radicals stimulated by oxLDL, we utilized the DHE fluorescence probe and H_2_O_2_-specific fluorescence probe (BES-H_2_O_2_-Ac) to measure the content of reactive oxygen species (ROS) in THP-1-derived human macrophages. The results obtained from the DHE fluorescence probe revealed a significant increase in O_2_^−^ content in the oxLDL group compared to the control group. However, no statistical difference in the content of O_2_^−^ was observed between the oxLDL + L-Cth 0.1 mM group and oxLDL group. Notably, both 0.3 mM and 1.0 mM L-Cth treatment exhibited an inhibitory effect on the generation of O_2_^−^ in the macrophage induced by oxLDL (Figure 1A).

Similarly, the results obtained from the H_2_O_2_ fluorescence probe demonstrated a prominent enhancement of H_2_O_2_ content in the oxLDL group compared to the control group. In contrast, the oxLDL + L-Cth 0.1 mM group showed no significant difference in H_2_O_2_ production when compared to the oxLDL group. However, the oxLDL + L-Cth 0.3 mM and oxLDL + L-Cth 1.0 mM groups exhibited a notable decrease in H_2_O_2_ production (Figure 1B).

### 3.2 L-Cth inhibited oxLDL-stimulated iNOS protein expression and NO production in THP-1 monocyte-derived human macrophages

**FIGURE 2:**
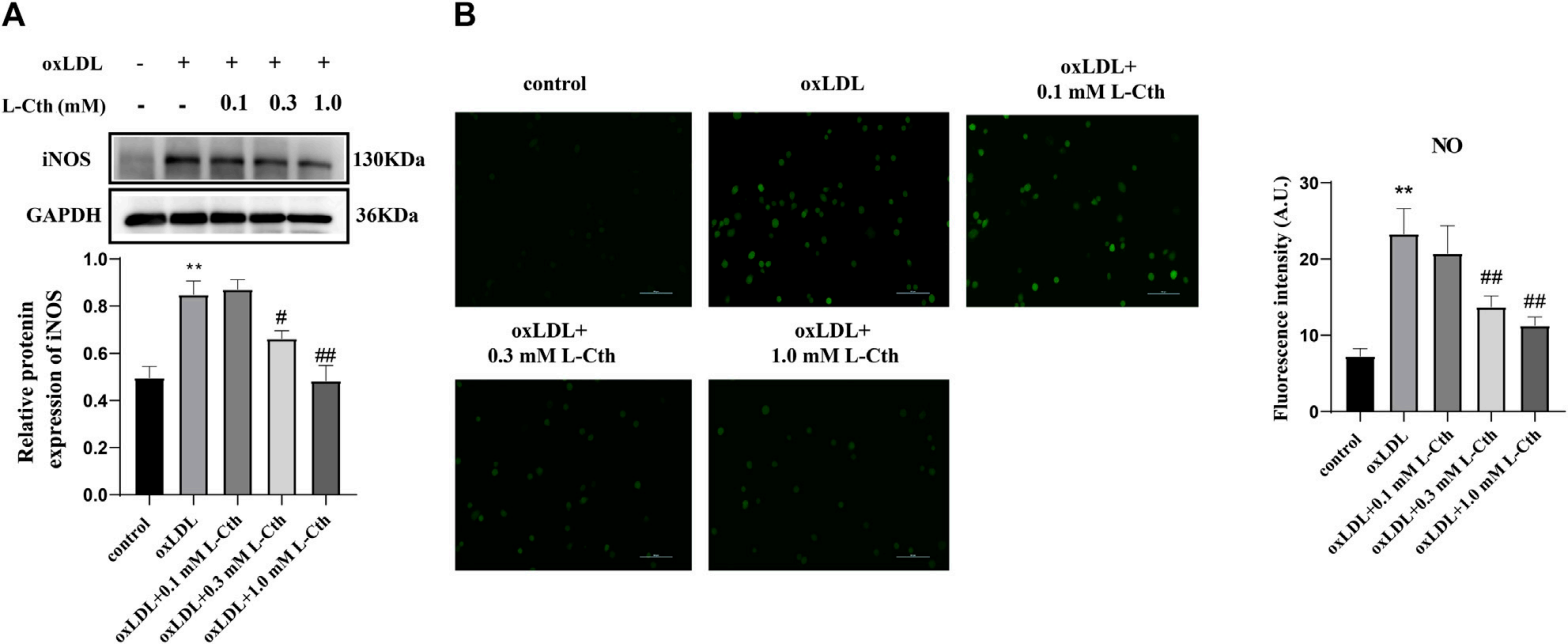
L-Cth inhibited oxLDL-stimulated iNOS protein expression and NO production in human macrophages. **(A)** iNOS protein expression detected by Western blotting. **(B)** NO content in each group was detected using a fluorescence probe. These data were from three independent cultures. **p < 0.01, vs. control group; ^##^ *p* < 0.01, ^#^ *p* < 0.05, vs. oxLDL group.

In addition to detecting reactive oxygen species, we also assessed the expression of iNOS protein and the NO content to investigate whether L-Cth could inhibit the production of reactive nitrogen species (RNS). The results showed that the iNOS protein level in the cells of oxLDL group was increased compared to the control group (*p* < 0.01) (Figure 2A). There was no significant difference in iNOS expression between the oxLDL + L-Cth 0.1 mM group and the oxLDL group. However, iNOS expression was significantly decreased in both the oxLDL + L-Cth 0.3 mM group and the oxLDL + L-Cth 1.0 mM group compared to the oxLDL group (*p* < 0.05 and *p* < 0.01, respectively).

Moreover, we utilized a nitric oxide fluorescence probe to specifically measure the production of NO in each group. Consistent with the previous findings, the fluorescence intensity was markedly increased in the oxLDL-treated group, indicating an elevation in NO production. There was no significant change of the green fluorescence intensity between the oxLDL + L-Cth 0.1 mM group and the oxLDL group. However, the NO production in cells pretreated with 0.3 mM L-Cth and 1.0 mM L-Cth was visibly reduced (Figure 2B).

### 3.3 L-Cth antagonized inhibitory effect of oxLDL on GSH-Px, SOD, and CAT activity in THP-1 monocyte-derived human macrophages

**FIGURE 3:**
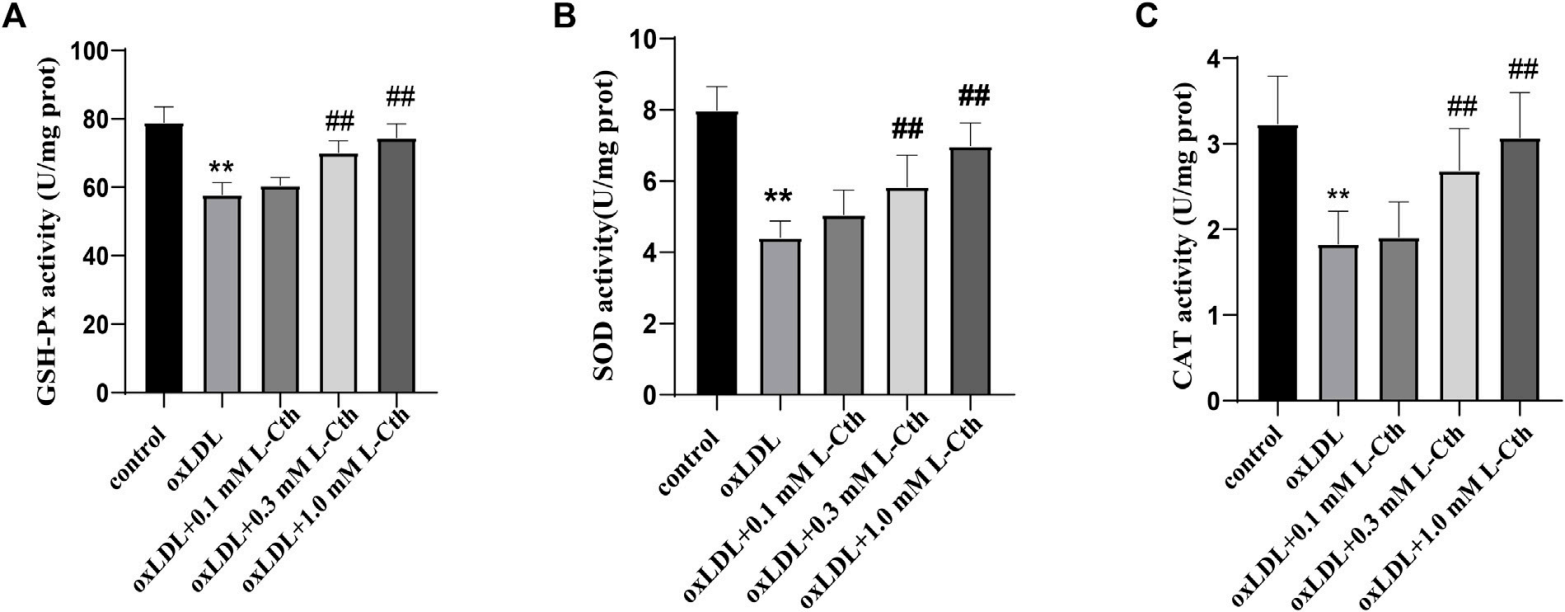
L-Cth antagonized inhibitory effect of oxLDL on GSH-Px, SOD, and CAT activity in THP-1 monocyte-derived human macrophages. **(A)** GSH-Px activity detected by chemical colorimetry. **(B)** SOD activity detected by chemical colorimetry. **(C)** CAT activity detected by chemical colorimetry. These data were from three independent cultures. **p < 0.01, vs. control group; ^##^ *p* < 0.01, vs. oxLDL group.

The results demonstrated that oxLDL significantly reduced the activity of GSH-Px, SOD, and CAT by 26.8%, 44.8%, and 43.4%, respectively (all *p* < 0.01) (Figures 3A–C). In contrast, the GSH-Px, SOD, and CAT activity in the cells of the oxLDL + L-Cth 0.1 mM group did not show significant changes. However, pre-treatment with 0.3 mM L-Cth resulted in an increase by 21.4% in GSH-Px activity, 32.4% in SOD activity, and 47.1% in CAT activity (all *p* < 0.01). Moreover, pretreatment with 1 mM L-Cth elevated GSH-Px, SOD, and CAT activity by 28.9%, 58.4%, and 68.0%, respectively (all *p* < 0.01). These findings indicate that L-Cth can counteract the inhibitory effect of oxLDL on GSH-Px, SOD, and CAT activity in THP-1-derived human macrophages.

### 3.4 L-Cth increased the SOD1 and SOD2 protein expression downregulated by oxLDL in THP-1 monocyte-derived human macrophages

**FIGURE 4:**
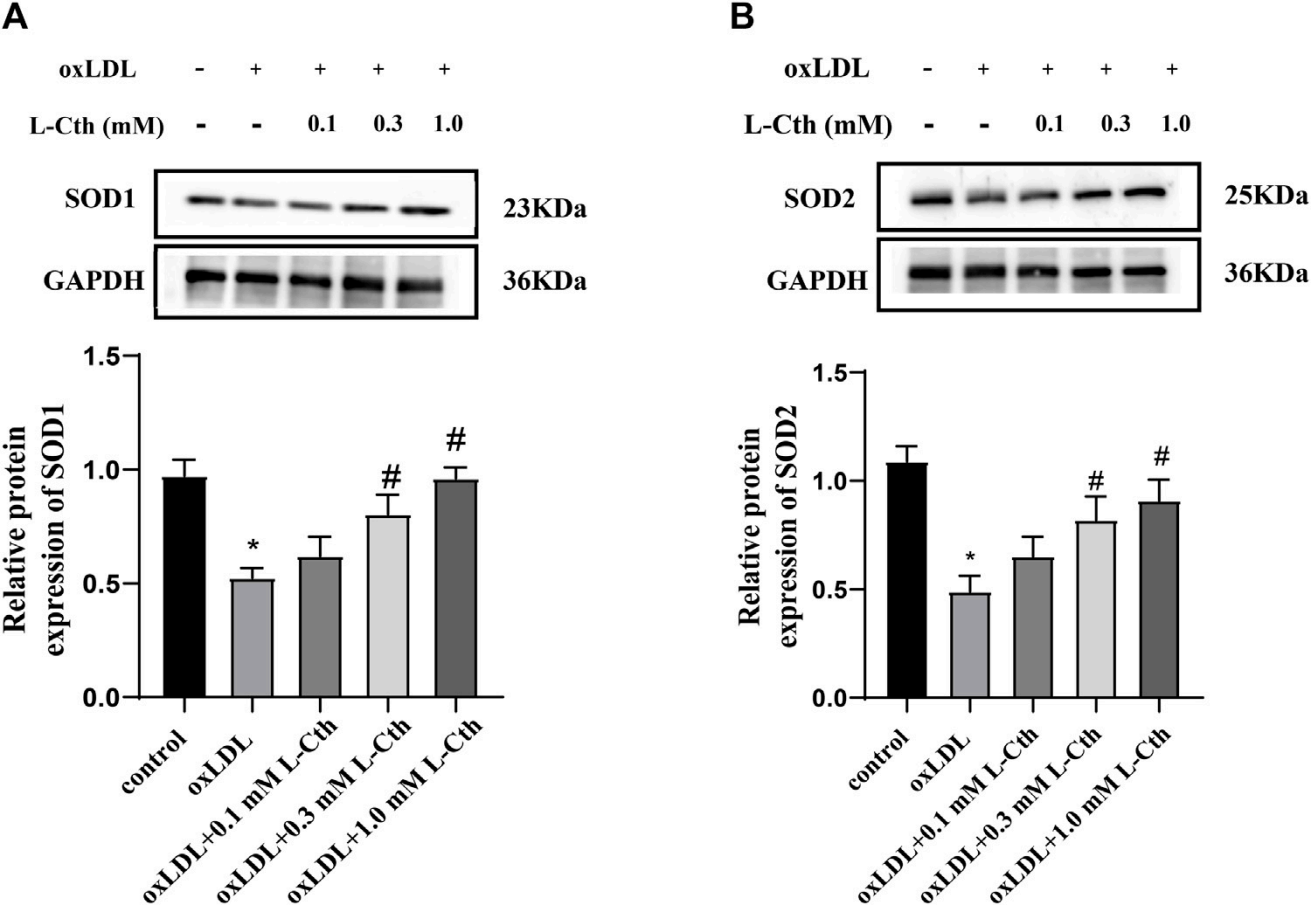
L-Cth increased the SOD1 and SOD2 protein expression downregulated by oxLDL in THP-1 monocyte-derived human macrophages. **(A)** SOD1 expression detected by Western blotting. **(B)** SOD2 expression detected by Western blotting. These data were from three independent cultures. *p < 0.05, vs. control group; ^#^ *p* < 0.05, vs. oxLDL group.

The results demonstrated that the protein expressions of SOD1 and SOD2 were significantly suppressed by oxLDL compared to the control group (*p* < 0.01, *p* < 0.01) (Figures 4A,B). In contrast, in the oxLDL + L-Cth 0.1 mM group, there was no significant alteration in the expressions of SOD1 and SOD2 proteins. However, the protein expressions of SOD1 and SOD2 were significantly elevated in the oxLDL + L-Cth 0.3 mM group (*p* < 0.01, *p* < 0.01) and the oxLDL + L-Cth 1.0 mM group (*p* < 0.01, *p* < 0.01), respectively. These findings suggest that L-Cth can enhance the protein expression of SOD1 and SOD2 in human macrophages stimulated by oxLDL.

### 3.5 L-Cth antagonized DNA damage caused by oxLDL in THP-1 monocyte-derived human macrophages

**FIGURE 5:**
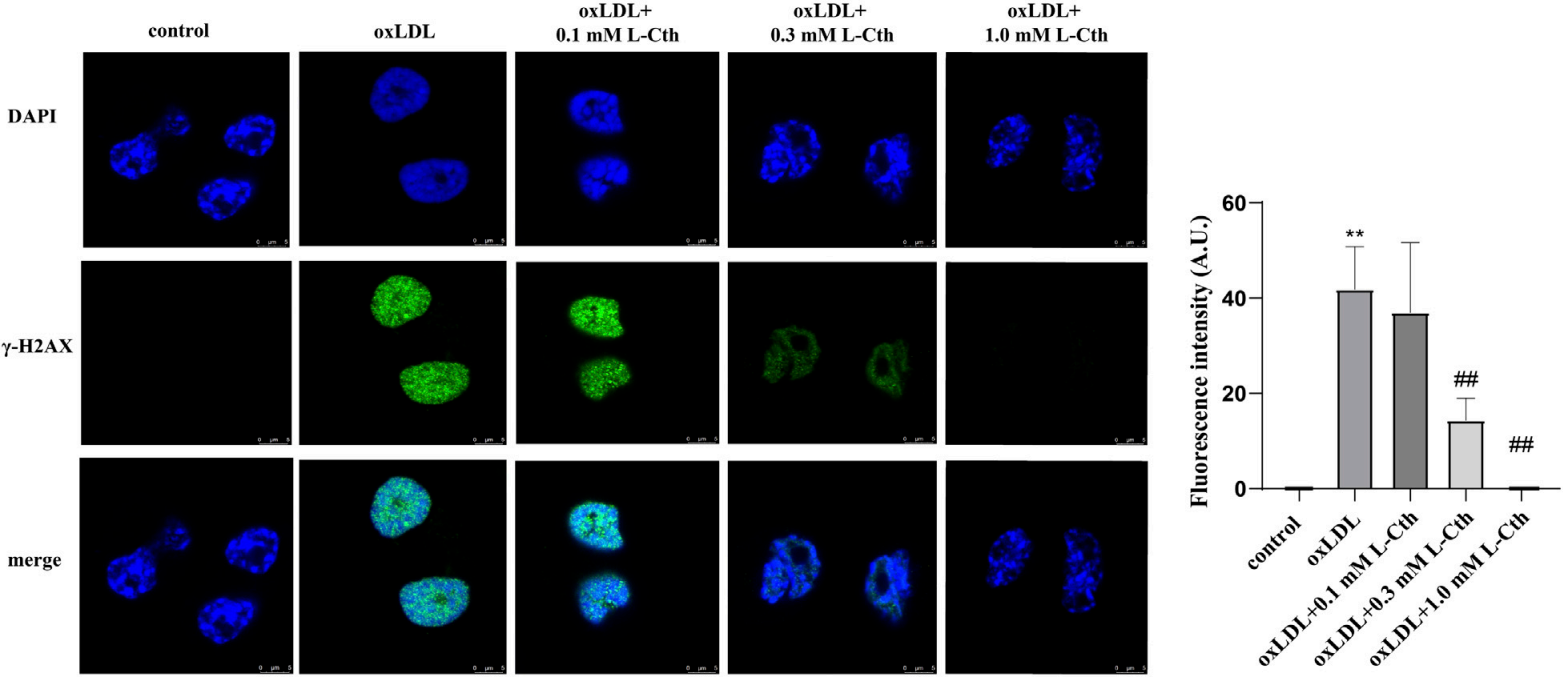
L-Cth reversed DNA damage caused by oxLDL in THP-1 monocyte-derived human macrophages. γ-H2AX (phosphorylated H2AX) immunofluorescence was used to measure DNA damage in human macrophages in each group. These data were from three independent cultures. **p < 0.01, vs. control group; ^##^ *p* < 0.01, vs. oxLDL group.

To examine the impact of L-Cth on DNA damage in human macrophages, γ-H2AX immunofluorescence was employed as a measure of DNA damage across the experimental groups. The fluorescence intensity of γ-H2AX in the cells of oxLDL group was notably enhanced compared to the control group, indicating a substantial DNA damage. However, no significant difference in γ-H2AX fluorescence intensity was observed between the cells in the oxLDL + L-Cth 0.1 mM group and the oxLDL group, suggesting that DNA damage remained largely unchanged. In contrast, cells in the oxLDL + L-Cth 0.3 mM and oxLDL + L-Cth 1.0 mM groups exhibited a substantial reduction in fluorescence intensity, indicating a significant decrease in DNA damage (Figure 5). These findings suggest that L-Cth effectively inhibits DNA damage induced by oxLDL in human macrophages.

### 3.6 Activation of cystathionine-β-synthase (CBS) recapitulated the effect of L-Cth on cell protection, while inhibition of CBS exacerbated the detrimental effect of oxLDL

**FIGURE 6:**
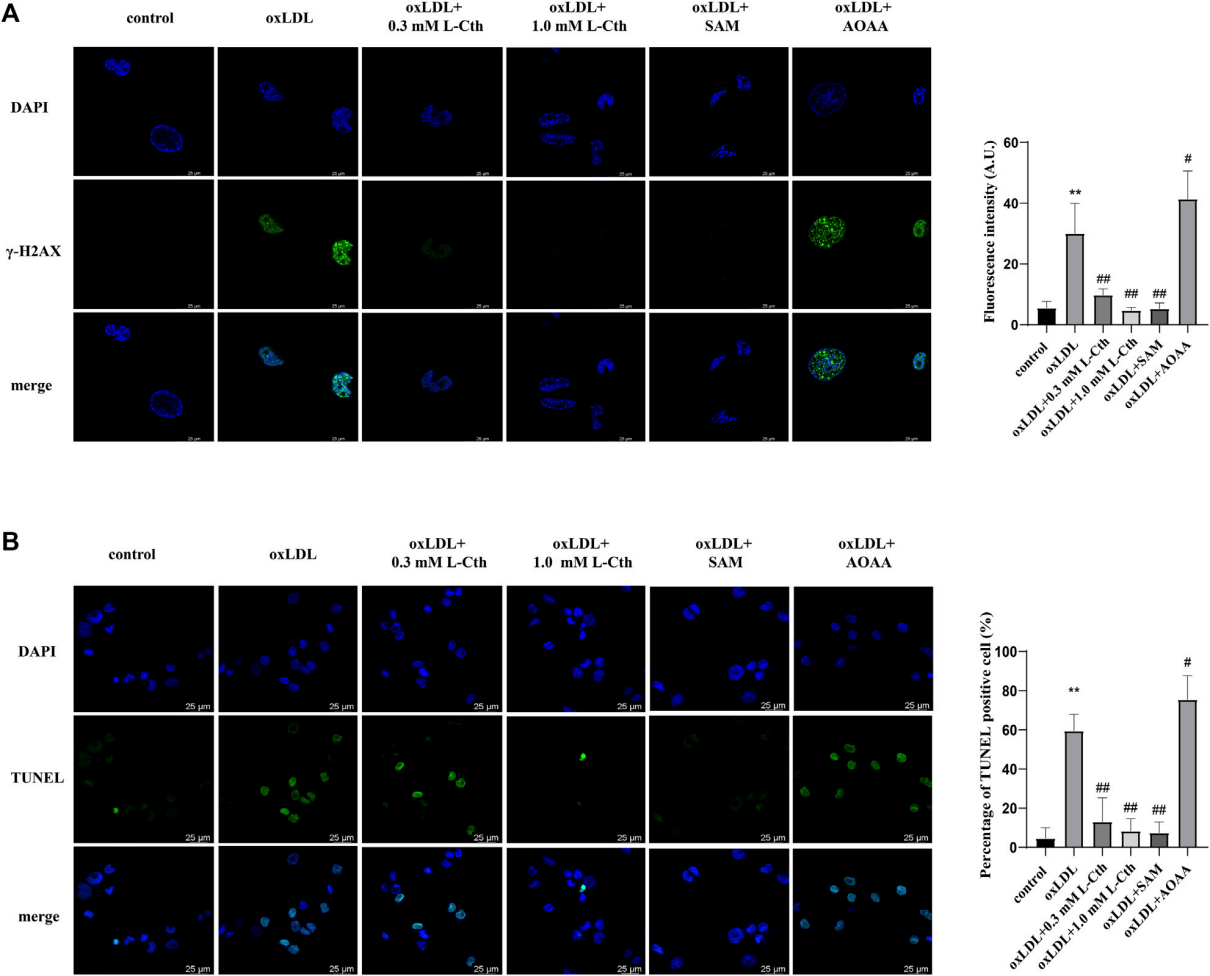
Activation of CBS recapitulated the effect of L-Cth on cell protection, while inhibition of CBS exacerbated the detrimental effect of oxLDL. **(A)** γ-H2AX immunofluorescence was used to measure DNA damage in each group. **(B)** TUNEL assay was used to detect the apoptosis of cells. These data were from three independent cultures. **p < 0.01, vs. control group; ^##^ *p* < 0.01, ^#^ *p* < 0.05 vs. oxLDL group.

To further substantiate the crucial role of L-Cth in cellular protection, we interfered with the endogenous production of L-Cth using an allosteric activator of CBS (SAM) and an inhibitor of CBS (AOAA). As anticipated, pre-treatment with SAM significantly mitigated the oxLDL-induced DNA damage and cell apoptosis in THP-1-derived macrophages, while pre-treatment with AOAA exacerbated the abovementioned cell injury by oxLDL (Figure 6). These findings indicate that the activation of endogenous L-Cth production recapitulated the effect observed with exogenous L-Cth on cellular protection, whereas the inhibition of endogenous L-Cth production exacerbated the detrimental impact of oxLDL.

### 3.7 The cytoprotective effect of L-Cth might not be mediated by H_2_S

**FIGURE 7:**
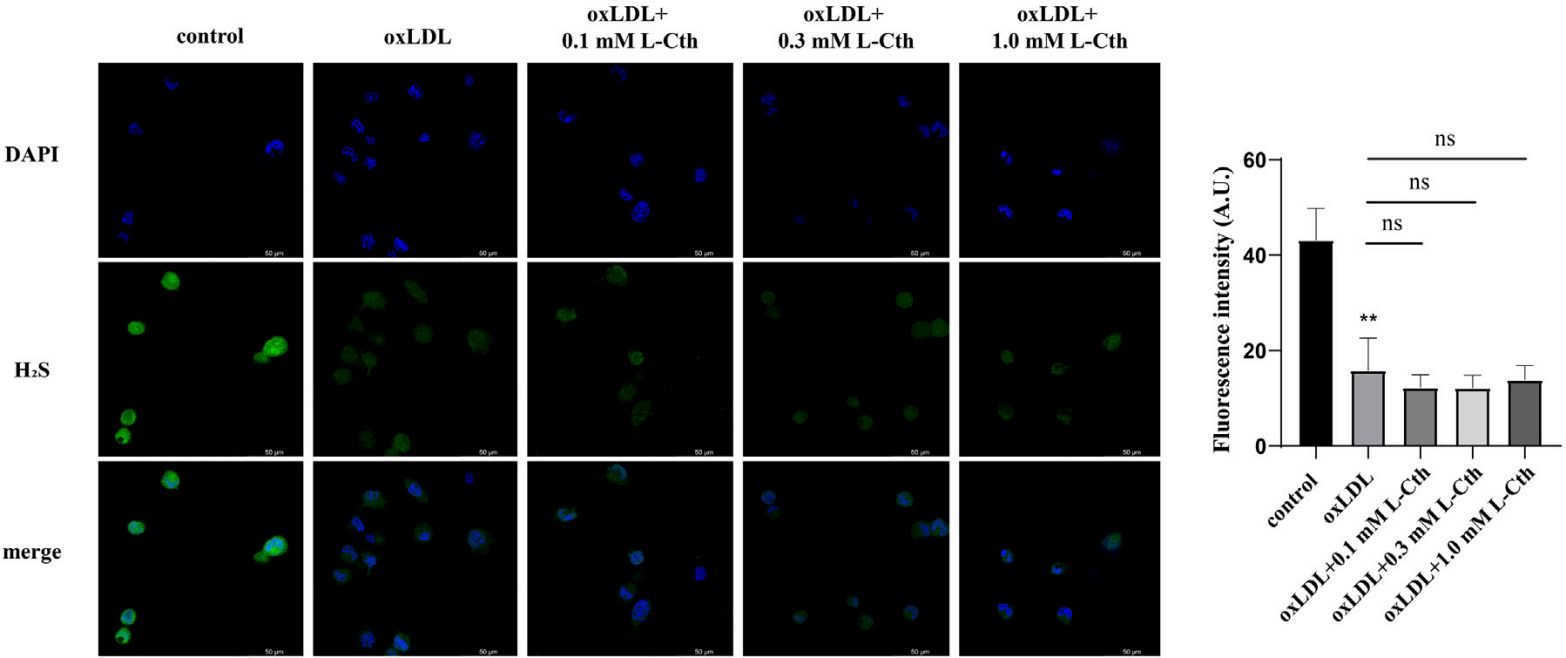
Treatment with L-Cth did not change the production of H_2_S in oxLDL-stimulated macrophages. A fluorescent sulfide specific probe was used to detect the H_2_S content in THP-1-derived macrophages treated with oxLDL and L-Cth. These data were from three independent cultures. **p < 0.01, vs. control group; ns, not significant.

Considering that L-Cth can undergo further metabolism into H_2_S through CSE-mediated enzymatic reactions (Lv et al., 2021), we utilized a fluorescent H_2_S-specific probe to investigate the impact of L-Cth on endogenous H_2_S production in macrophages stimulated with oxLDL. The results revealed a significant reduction in the intensity of H_2_S fluorescence following oxLDL treatment. However, pre-treatment with L-Cth did not alter H_2_S fluorescence intensity in oxLDL-injured cells (Figure 7). These findings suggest that H_2_S might not be correlated with the protective effect of L-Cth against oxLDL-induced cell injury.

## 4 Discussion

The present study demonstrates that L-Cth effectively inhibits oxidative stress induced by oxLDL in macrophages by suppressing the production of free radicals and enhancing the antioxidant capacity. Furthermore, L-Cth attenuates oxLDL-induced DNA damage and apoptosis in monocyte-derived macrophages.

In mammals, L-Cth serves as an intermediate metabolite in the metabolic pathway of sulfur-containing amino acids. Methionine is bio-converted to S-adenosyl-methionine by methionine adenosyl transferase, and then the latter is demethylated to produce S-adenosine homocysteine. S-adenosine homocysteine is subsequently hydrolyzed to homocysteine (Hcy). Catalyzed by CBS, Hcy and serine are converted to L-Cth. Then, L-Cth is decomposed into cysteine, α-ketobutyric acid and ammonium ions catalyzed by cystathionine-γ-lyase (CSE) (Klein et al., 1988). Research shows that the expression of CBS in human monocyte macrophages is downregulated under the inflammatory conditions induced by oxLDL, suggesting the potential involvement of L-Cth, as a catalytic product of CBS, in the mechanism of oxLDL-induced macrophage injury (Du et al., 2014). Wada *et al.* demonstrated that L-Cth reduces the production of superoxide radicals by human leukocytes *in vitro* in a dose-dependent manner (Wada et al., 1996). Other studies have shown that L-Cth inhibited U937 and HepG2 apoptosis via keeping from GSH excretion (Ghibelli et al., 1998). However, the precise mechanism by which L-Cth regulates oxidative stress in monocyte-derived macrophages during atherosclerosis remains unclear. Therefore, further investigation is necessary to elucidate the potential regulatory mechanisms underlying the effect of L-Cth on oxidative stress in monocyte-derived macrophages.

Excessive production of reactive oxygen/nitrogen species and/or compromised antioxidant capacity of macrophages can lead to an imbalanced redox state, ultimately resulting in atherosclerotic vascular damage (Kattoor et al., 2017). Research has revealed that extracts from atherosclerotic lesions promote atherosclerosis development by increasing oxidative stress and lipid accumulation in macrophages (Abu-Saleh et al., 2016). Inflammatory conditions and other factors induce the expression of iNOS, leading to the release of nitric oxide (NO). NO can react with O_2_^−^ to form RNS such as peroxynitrite and other secondary products including NO_2_^+^, NO_2_, and -OH, triggering cascade redox reactions (Wieronska et al., 2021). ROS and RNS may primarily exert the effects through interactions with specific proteins containing iron-sulfur clusters and cysteine sulfhydryl groups (Lushchak and Lushchak, 2021). O_2_^−^ is a major source of ROS and, when combined with -OH, it results in DNA damage and is closely associated with various inflammatory diseases. H_2_O_2_, another significant ROS, disrupts mitochondrial and cellular membrane integrity through lipid peroxidation, causing tissue damage and serving as a marker of oxidative stress damage. Targeting H_2_O_2_ scavenging nanomaterials effectively eliminate abundant reactive oxygen species in atherosclerotic lesions, thus reducing oxidative stress and potentially offering therapeutic benefits for atherosclerosis (Liang et al., 2022). iNOS is considered to be a pathological form of nitric oxide synthase as it generates large amounts of NO and reacts with H_2_O_2_ to promote highly reactive hydroxyl radical (-OH) production (Wieronska et al., 2021). In this study, we observed that L-Cth dose-dependently mitigated oxidative stress in human macrophages stimulated by oxLDL. Notably, L-Cth reduced O_2_^−^ and H_2_O_2_ levels, iNOS protein expression, and NO content induced by iNOS, indicating that L-Cth significantly counteracted oxidative stress triggered by oxLDL in THP-1 monocyte-derived human macrophages.

The antioxidant system comprises enzymes and antioxidants, including GSH-Px, SOD, CAT, and GSH. GSH-Px serves as a critical peroxidase that converts toxic peroxides into non-toxic hydroxyl compounds, thus protecting cells from peroxide-induced damage. SOD, another important antioxidant enzyme, converts naturally occurring but harmful superoxide radicals into peroxides and H_2_O_2_, which are subsequently decomposed into harmless water by GSH-Px and CAT (Sinha et al., 2013; Poznyak et al., 2020b). GSH, an intracellular antioxidant, directly and indirectly eliminates free radicals to safeguard cells against oxidative stress. The antioxidant system forms the basis for resistance against free radicals, and therefore, antioxidant therapy is commonly employed in the treatment of atherosclerosis. Antioxidants such as angiotensin receptor antagonists, vitamins, calcium antagonists, angiotensin-converting enzyme inhibitors, and statins have been reported to inhibit oxidative stress (Yang et al., 2017). In the present study, we observed that L-Cth treatment dose-dependently increased the activities of GSH-Px, SOD, and CAT, as well as the protein expressions of SOD1 and SOD2 in macrophages stimulated by oxLDL. These results suggest that these antioxidant enzymes may contribute to the antioxidant effect of L-Cth. Moreover, L-Cth can be metabolized into L-cysteine by CSE, and L-cysteine can then be incorporated into GSH (Robert et al., 2005; Jurkowska et al., 2011). L-Cth supplementation has been shown to increase GSH levels in the liver, small intestine, and gastrocnemius muscle of healthy aged rats (Pouget et al., 2016). These observations imply that GSH might also play a role in the antioxidant effect of L-Cth. Hence, we speculate that the antioxidant properties of L-Cth may be attributed to the activation of antioxidative enzymes, including SOD, CAT, and GSH-Px, the upregulation of SOD1/2 protein expression, and the enhancement of GSH production.

Sustained ROS production can lead to oxidative damage, including DNA breakage protein denaturation, and lipid peroxidation (Flaherty et al., 2017; Jie et al., 2022). ROS acts as a crucial mediator of DNA damage, which can occur through direct oxidation of nucleoside bases and influences on the cell cycle (Srinivas et al., 2019). Excessive ROS production has been found to cause DNA breakage in the apoE^−/−^ mouse model of spontaneous atherosclerosis (Srinivas et al., 2019). Furthermore, in THP-1-derived macrophages, oxLDL treatment has been shown to elevate the levels of 8-hydroxydeoxyguanosine and significantly increase comet tail length, indicative of severe DNA damage (Jacinto et al., 2018; Zhang et al., 2018). DNA double-strand breaks are associated with increased expression of phosphorylated histone H2AX, specifically phosphorylated at Ser139 (γ-H2AX), which serves as a crucial marker for detecting DNA damage (Sedelnikova et al., 2003; Sharma et al., 2012). In this study, we employed γ-H2AX immunofluorescence to observe oxLDL-induced DNA damage in monocyte-derived macrophages. The results revealed that oxLDL treatment significantly enhanced γ-H2AX fluorescence intensity, while pre-treatment with L-Cth attenuated DNA damage in the oxLDL-injured THP-1-derived human macrophages. These findings suggest that L-Cth might protect macrophages against DNA damage triggered by oxLDL.

In addition to DNA damage, oxLDL treatment has been shown to induce macrophage apoptosis (Sun et al., 2018). TUNEL assay is a classical method to detect cell apoptosis by labeling the blunt ends of double-stranded DNA breaks (Kyrylkova et al., 2012). Consistent with the protective effect of exogenous L-Cth supplementation on cells, pre-treatment with a CBS activator to increase endogenous L-Cth production attenuated oxLDL-induced DNA damage and cell apoptosis in THP-1-derived macrophages. Conversely, pre-treatment with a CBS inhibitor to reduce endogenous L-Cth production exacerbated the detrimental effects of oxLDL, further confirming the cytoprotective effect of L-Cth.

Given that L-Cth can be metabolized into H_2_S through a CSE-mediated enzymatic reaction under physiological conditions (Huang et al., 2021), it is plausible that L-Cth treatment could upregulate endogenous H_2_S production in macrophages. H_2_S plays a crucial role in the regulation of macrophage functions such as chemotaxis, activation, and apoptosis (Zhang et al., 2023). Interestingly, in our study, we found that L-Cth did not increase H_2_S-specific fluorescence intensity in macrophages under oxLDL-stimulated conditions, other than under physiological conditions. This discrepancy may be attributed to the inhibition of the CSE/H_2_S pathway by oxLDL (Wang et al., 2013), which hampers the upregulation of H_2_S production by L-Cth. Consequently, those abovementioned results suggest that endogenous H_2_S might not be involved in the protective effect of L-Cth against oxLDL-induced cell injury, despite previous reports indicating a protective role of H_2_S in macrophages exposed to oxLDL.

Furthermore, the mechanism by which L-Cth is taken up into macrophages remains unclear. Previous studies have shown that L-Cth can be transported into immune tissue via the cystine/glutamate transporter (Kobayashi et al., 2015). Cystine/glutamate transporter was reported to be expressed in the macrophages and the level of ROS was significantly increased in cystine/glutamate transporter-deficient macrophages (Kobayashi et al., 2018). Moreover, cystine/glutamate transporter inhibitors, such as S-4-carboxyphenylglycine and glutamate, markedly promoted the macrophage oxidative damage and cell death (Pfau et al., 2012). Therefore, we speculate that L-Cth may enter macrophages through the cystine/glutamate transporter to exert its cytoprotective effects.

In summary, L-Cth effectively upregulates the capacity of antioxidant enzymes as well as the protein expression of SOD1/2, while reducing O_2_^−^ and H_2_O_2_ levels, iNOS-induced NO production, and oxidative DNA damage in macrophages stimulated by oxLDL. However, the precise underlying mechanisms by which L-Cth acts as an antioxidant require further investigation. Additionally, the protective effect of L-Cth against oxLDL-induced DNA damage should be further validated in animal models. This study uncovers the antioxidant effects of L-Cth in atherosclerosis, laying the foundation for future research on other free-radical-related cardiovascular diseases. These findings suggest that L-Cth could emerge as a promising target for the prevention and treatment of oxidative stress-related cardiovascular diseases.

## Data Availability

The original contributions presented in the study are included in the article/Supplementary Material, further inquiries can be directed to the corresponding authors.

## Abbreviations

AOAA, aminooxy acetic acid; CAT, catalase; CBS, cystathionine-β-synthase; CSE, cystathionine-γ-lyase; DHE, dihydroethidium; γ-H2AX, phosphorylated H2A histone family member X; GSH-Px, glutathione peroxidase; H_2_O_2_, hydrogen peroxide; H_2_S, hydrogen sulfide; Hcy, homocysteine; L-Cth, L-cystathionine; iNOS, inducible nitric oxide synthase; NO, nitric oxide; O_2_^−^, superoxide anions; -OH, hydroxyl radicals; ONOO^−^, peroxynitrite; oxLDL, oxidized low density lipoprotein; PMA, phorbol 12-myristate 13-acetate; RNS, reactive nitrogen species; ROS, reactive oxygen species; SAM, S-adenoslymethionine; SOD, superoxide dismutase.

## References

[B1] Abu-Saleh N. Aviram M. Hayek T. (2016). Aqueous or lipid components of atherosclerotic lesion increase macrophage oxidation and lipid accumulation. Life Sci. 154, 1–14. 10.1016/j.lfs.2016.04.019 27114099

[B2] Bennett M. R. (2001). Reactive oxygen species and death - oxidative DNA damage in atherosclerosis. Circulation Res. 88 (7), 648–650. 10.1161/hh0701.089955 11304484

[B3] Bjorkegren J. L. M. Lusis A. J. (2022). Atherosclerosis: Recent developments. Cell 185 (10), 1630–1645. 10.1016/j.cell.2022.04.004 35504280 PMC9119695

[B4] Chen K. L. Li L. Li C. M. Wang Y. R. Yang F. X. Kuang M. Q. (2019). SIRT7 regulates lipopolysaccharide-induced inflammatory injury by suppressing the NF-*κ*B signaling pathway. Oxid. Med. Cell Longev. 2019, 3187972. 10.1155/2019/3187972 31285783 PMC6594283

[B5] Cominacini L. Garbin U. Mozzini C. Stranieri C. Pasini A. Solani E. (2015). The atherosclerotic plaque vulnerability: Focus on the oxidative and endoplasmic reticulum stress in orchestrating the macrophage apoptosis in the formation of the necrotic core. Curr. Med. Chem. 22 (13), 1565–1572. 10.2174/0929867322666150311150829 25760090

[B6] Du J. Huang Y. Li K. Yu X. Jin H. Yang L. (2018). Retina-derived endogenous sulfur dioxide might be a novel anti-apoptotic factor. Biochem. Biophys. Res. Commun. 496, 955–960. 10.1016/j.bbrc.2018.01.103 29402407

[B7] Du J. Huang Y. Yan H. Zhang Q. Zhao M. Zhu M. (2014). Hydrogen sulfide suppresses oxidized low-density lipoprotein (ox-LDL)-stimulated monocyte chemoattractant protein 1 generation from macrophages via the nuclear factor κB (NF-κB) pathway. J. Biol. Chem. 289 (14), 9741–9753. 10.1074/jbc.M113.517995 24550391 PMC3975021

[B8] Flaherty R. L. Owen M. Fagan-Murphy A. Intabli H. Healy D. Patel A. (2017). Glucocorticoids induce production of reactive oxygen species/reactive nitrogen species and DNA damage through an iNOS mediated pathway in breast cancer. Breast Cancer Res. 19, 35. 10.1186/s13058-017-0823-8 28340615 PMC5366114

[B9] Gaull G. Sturman J. A. Raiha N. C. R. (1972). Development of mammalian sulfur metabolism - absence of cystathionase in human fetal tissues. Pediatr. Res. 6 (6), 538–547. 10.1203/00006450-197206000-00002 4625813

[B10] Ghibelli L. Fanelli C. Rotilio G. Lafavia E. Coppola S. Colussi C. (1998). Rescue of cells from apoptosis by inhibition of active GSH extrusion. FASEB J. 12 (6), 479–486. 10.1096/fasebj.12.6.479 9535220

[B11] Huang Y. Shen Z. Chen Q. Huang P. Zhang H. Du S. (2016). Endogenous sulfur dioxide alleviates collagen remodeling via inhibiting TGF-β/Smad pathway in vascular smooth muscle cells. Sci. Rep. 6, 19503. 10.1038/srep19503 26762477 PMC4725894

[B12] Huang Y. Q. Jin H. F. Zhang H. Tang C. S. Du J. B. (2021). Interaction among hydrogen sulfide and other gasotransmitters in mammalian physiology and pathophysiology. Adv. Exp. Med. Biol. 1315, 205–236. 10.1007/978-981-16-0991-6_9 34302694

[B13] Jacinto T. A. Meireles G. S. Dias A. T. Aires R. Porto M. L. Gava A. L. (2018). Increased ROS production and DNA damage in monocytes are biomarkers of aging and atherosclerosis. Biol. Res. 51 (1), 33. 10.1186/s40659-018-0182-7 30185234 PMC6123971

[B14] Jie Z. S. Liu J. Shu M. C. Ying Y. Yang H. F. (2022). Detection strategies for superoxide anion: A review. Talanta 236, 122892. 10.1016/j.talanta.2021.122892 34635271

[B15] Jurkowska H. Placha W. Nagahara N. Wrobel M. (2011). The expression and activity of cystathionine-gamma-lyase and 3-mercaptopyruvate sulfurtransferase in human neoplastic cell lines. Amino Acids 41 (1), 151–158. 10.1007/s00726-010-0606-3 20446008

[B16] Kanzaki H. Shinohara F. Kajiya M. Fukaya S. Miyamoto Y. Nakamura Y. (2014). Nuclear Nrf2 induction by protein transduction attenuates osteoclastogenesis. Free Radic. Biol. Med. 77, 239–248. 10.1016/j.freeradbiomed.2014.09.006 25224039

[B17] Kattoor A. J. Pothineni N. V. K. Palagiri D. Mehta J. L. (2017). Oxidative stress in atherosclerosis. Curr. Atheroscler. Rep. 19 (11), 42. 10.1007/s11883-017-0678-6 28921056

[B18] Khatana C. Saini N. K. Chakrabarti S. Saini V. Sharma A. Saini R. V. (2020). Mechanistic insights into the oxidized low-density lipoprotein-induced atherosclerosis. Oxid. Med. Cell Longev. 2020, 5245308. 10.1155/2020/5245308 33014272 PMC7512065

[B19] Klein C. E. Roberts B. Holcenberg J. Glode L. M. (1988). Cystathionine metabolism in neuroblastoma. Cancer 62 (2), 291–298. 10.1002/1097-0142(19880715)62:2<291::aid-cncr2820620211>3.0.co;2-q 3383129

[B20] Kobayashi S. Hamashima S. Homma T. Sato M. Kusumi R. Bannai S. (2018). Cystine/glutamate transporter, system Xc^−^, is involved in nitric oxide production in mouse peritoneal macrophages. Nitric Oxide 78, 32–40. 10.1016/j.niox.2018.05.005 29792932

[B21] Kobayashi S. Sato M. Kasakoshi T. Tsutsui T. Sugimoto M. Osaki M. (2015). Cystathionine is a novel substrate of cystine/glutamate transporter: Implications for immune function. J. Biol. Chem. 290 (14), 8778–8788. 10.1074/jbc.M114.625053 25713140 PMC4423669

[B22] Kyrylkova K. Kyryachenko S. Leid M. Kioussi C. (2012). Detection of apoptosis by TUNEL assay. Methods Mol. Biol. 887, 41–47. 10.1007/978-1-61779-860-3_5 22566045

[B23] Li W. Shi C. Wu X. Zhang Y. Liu H. Wang X. (2022). Light activation of iridium(III) complexes driving ROS production and DNA damage enhances anticancer activity in A549 cells. J. Inorg. Biochem. 236, 111977. 10.1016/j.jinorgbio.2022.111977 36030672

[B24] Liang X. Li H. Li X. Tian X. Zhang A. Luo Q. (2022). Highly sensitive H_2_O_2_-scavenging nano-bionic system for precise treatment of atherosclerosis. Acta Pharm. Sin. B 13, 372–389. 10.1016/j.apsb.2022.04.002 36815039 PMC9939301

[B25] Lin V. S. Lippert A. R. Chang C. J. (2013). Cell-trappable fluorescent probes for endogenous hydrogen sulfide signaling and imaging H_2_O_2_-dependent H_2_S production. Proc. Natl. Acad. Sci. U. S. A. 110 (18), 7131–7135. 10.1073/pnas.1302193110 23589874 PMC3645565

[B26] Lushchak V. I. Lushchak O. (2021). Interplay between reactive oxygen and nitrogen species in living organisms. Chem. Biol. Interact. 349, 109680. 10.1016/j.cbi.2021.109680 34606757

[B27] Lv B. Chen S. Tang C. Jin H. Du J. Huang Y. (2021). Hydrogen sulfide and vascular regulation - an update. J. Adv. Res. 27, 85–97. 10.1016/j.jare.2020.05.007 33318869 PMC7728588

[B28] Peng H. Zhang S. Zhang Z. Wang X. Tian X. Zhang L. (2022). Nitric oxide inhibits endothelial cell apoptosis by inhibiting cysteine-dependent SOD1 monomerization. FEBS Open Bio 12 (2), 538–548. 10.1002/2211-5463.13362 PMC8804620 34986524

[B29] Pfau J. C. Seib T. Overocker J. J. Roe J. Ferro A. S. (2012). Functional expression of system x(c)- is upregulated by asbestos but not crystalline silica in murine macrophages. Inhal. Toxicol. 24 (8), 476–485. 10.3109/08958378.2012.689782 22697888 PMC3539215

[B30] Pouget M. Perrot M. Denis P. Vuichoud J. Dardevet D. Vidal K. (2016). Long-term dietary supplementation with cystathionine improves tissue glutathione in ageing rats. Aging Clin. Exp. Res. 28 (4), 781–785. 10.1007/s40520-015-0465-6 26514972

[B31] Poznyak A. V. Grechko A. V. Orekhova V. A. Chegodaev Y. S. Wu W. K. Orekhov A. N. (2020b). Oxidative stress and antioxidants in atherosclerosis development and treatment. Biol. (Basel) 9 (3), 60. 10.3390/biology9030060 PMC7150948 32245238

[B32] Poznyak A. V. Nikiforov N. G. Markin A. M. Kashirskikh D. A. Myasoedova V. A. Gerasimova E. V. (2020a). Overview of OxLDL and its impact on cardiovascular health: Focus on atherosclerosis. Front. Pharmacol. 11, 613780. 10.3389/fphar.2020.613780 33510639 PMC7836017

[B33] Robert K. Nehmé J. Bourdon E. Pivert G. Friguet B. Delcayre C. (2005). Cystathionine beta synthase deficiency promotes oxidative stress, fibrosis, and steatosis in mice liver. Gastroenterology 128 (5), 1405–1415. 10.1053/j.gastro.2005.02.034 15887121

[B34] Sedelnikova O. A. Pilch D. R. Redon C. Bonner W. M. (2003). Histone H2AX in DNA damage and repair. Cancer Biol. Ther. 2 (3), 233–235. 10.4161/cbt.2.3.373 12878854

[B35] Sharma A. Singh K. Almasan A. (2012). Histone H2AX phosphorylation: A marker for DNA damage. Methods Mol. Biol. 920, 613–626. 10.1007/978-1-61779-998-3_40 22941631

[B36] Sinha K. Das J. Pal P. B. Sil P. C. (2013). Oxidative stress: The mitochondria-dependent and mitochondria-independent pathways of apoptosis. Arch. Toxicol. 87 (7), 1157–1180. 10.1007/s00204-013-1034-4 23543009

[B37] Soehnlein O. Libby P. (2021). Targeting inflammation in atherosclerosis - from experimental insights to the clinic. Nat. Rev. Drug Discov. 20 (8), 589–610. 10.1038/s41573-021-00198-1 33976384 PMC8112476

[B38] Srinivas U. S. Tan B. W. Q. Vellayappan B. A. Jeyasekharan A. D. (2019). ROS and the DNA damage response in cancer. Redox Biol. 25, 101084. 10.1016/j.redox.2018.101084 30612957 PMC6859528

[B39] Sun W. Lin Y. Chen L. Ma R. Cao J. Yao J. (2018). Legumain suppresses OxLDL-induced macrophage apoptosis through enhancement of the autophagy pathway. Gene 652, 16–24. 10.1016/j.gene.2018.02.012 29414692

[B40] Wada K. Kamisaki Y. Kitano M. Nakamoto K. Itoh T. (1995). Protective effect of cystathionine on acute gastric mucosal injury induced by ischemia-reperfusion in rats. Eur. J. Pharmacol. 294 (2-3), 377–382. 10.1016/0014-2999(95)00558-7 8750697

[B41] Wada K. Kamisaki Y. Nakamoto K. Itoh T. (1996). Effect of cystathionine as a scavenger of superoxide generated from human leukocytes or derived from xanthine oxidase *in vitro* . Eur. J. Pharmacol. 296 (3), 335–340. 10.1016/0014-2999(95)00717-2 8904086

[B42] Wang X. H. Wang F. You S. J. Cao Y. J. Cao L. D. Han Q. (2013). Dysregulation of cystathionine γ-lyase (CSE)/hydrogen sulfide pathway contributes to ox-LDL-induced inflammation in macrophage. Cell Signal 25 (11), 2255–2262. 10.1016/j.cellsig.2013.07.010 23872072

[B43] Wei L. Lu N. Dai Q. Rong J. Chen Y. Li Z. (2010). Different apoptotic effects of wogonin via induction of H(2)O(2) generation and Ca(2+) overload in malignant hepatoma and normal hepatic cells. J. Cell Biochem. 111 (6), 1629–1641. 10.1002/jcb.22898 21053277

[B44] Wieronska J. M. Cieslik P. Kalinowski L. (2021). Nitric oxide-dependent pathways as critical factors in the consequences and recovery after brain ischemic hypoxia. Biomolecules 11 (8), 1097. 10.3390/biom11081097 34439764 PMC8392725

[B45] Xu H. Jiang J. Chen W. Li W. Chen Z. (2019). Vascular macrophages in atherosclerosis. J. Immunol. Res. 2019, 4354786. 10.1155/2019/4354786 31886303 PMC6914912

[B46] Yang X. Y. Li Y. Li Y. D. Ren X. M. Zhang X. Y. Hu D. (2017). Oxidative stress-mediated atherosclerosis: Mechanisms and therapies. Front. Physiology 8, 600. 10.3389/fphys.2017.00600 PMC5572357 28878685

[B47] Zhang H. Du J. Huang Y. Tang C. Jin H. (2023). Hydrogen sulfide regulates macrophage function in cardiovascular diseases. Antioxid. Redox Signal 38 (1-3), 45–56. 10.1089/ars.2022.0075 35658575

[B48] Zhang J. Q. Shen M. Zhu C. C. Yu F. X. Liu Z. Q. Ally N. (2014b). 3-Nitropropionic acid induces ovarian oxidative stress and impairs follicle in mouse. PLoS One 9 (2), e86589. 10.1371/journal.pone.0086589 24505260 PMC3914797

[B49] Zhang M. Feng L. Gu J. Ma L. Qin D. Wu C. (2014a). The attenuation of Moutan Cortex on oxidative stress for renal injury in AGEs-induced mesangial cell dysfunction and streptozotocin-induced diabetic nephropathy rats. Oxid. Med. Cell Longev. 2014, 463815. 10.1155/2014/463815 24876912 PMC4021834

[B50] Zhang Q. Zeng X. K. Guo J. X. Wang M. (2002). Oxidant stress mechanism of homocysteine potentiating Con A-induced proliferation in murine splenic T lymphocytes. Cardiovasc Res. 53 (4), 1035–1042. 10.1016/s0008-6363(01)00541-7 11922914

[B51] Zhang Y. Wang C. Jin Y. Yang Q. Meng Q. Liu Q. (2018). Activating the PGC-1*α*/TERT pathway by catalpol ameliorates atherosclerosis via modulating ROS production, DNA damage, and telomere function: Implications on mitochondria and telomere link. Oxid. Med. Cell Longev. 2018, 2876350. 10.1155/2018/2876350 30046372 PMC6036816

[B52] Zhu M. Du J. Chen S. Liu A. D. Holmberg L. Chen Y. (2014). L-cystathionine inhibits the mitochondria-mediated macrophage apoptosis induced by oxidized low density lipoprotein. Int. J. Mol. Sci. 15 (12), 23059–23073. 10.3390/ijms151223059 25514411 PMC4284754

