# Supplementary material for: L-cystathionine protects against oxidative stress and DNA damage induced by oxidized low-density lipoprotein in THP-1-derived macrophages
Source: Front Pharmacol. 2023 Jul 25;14:1161542. doi: 10.3389/fphar.2023.1161542 (PMC10408194; doi:10.3389/fphar.2023.1161542)
Supplement: Supplementary file 1 [file DataSheet1.PDF]

Supplementary Materials

Supplementary Table 1: A list of chemicals employed in this study and from where they were purchased.

**Chemicals**	**Company**
AOAA	Selleck, USA
GAPDH antibody	Shanghai Kangcheng, China
iNOS antibody	CST, USA
L-cystathionine	Sigma, USA
oxLDL	Zhongshan Golden Bridge, China
PMA	Sigma, USA
SAM	Macklin, China
SOD1 antibody	Enzo, USA
SOD2 antibody	Enzo, USA

Supplementary Figure 1: The western blot analysis with molecular weight marker.

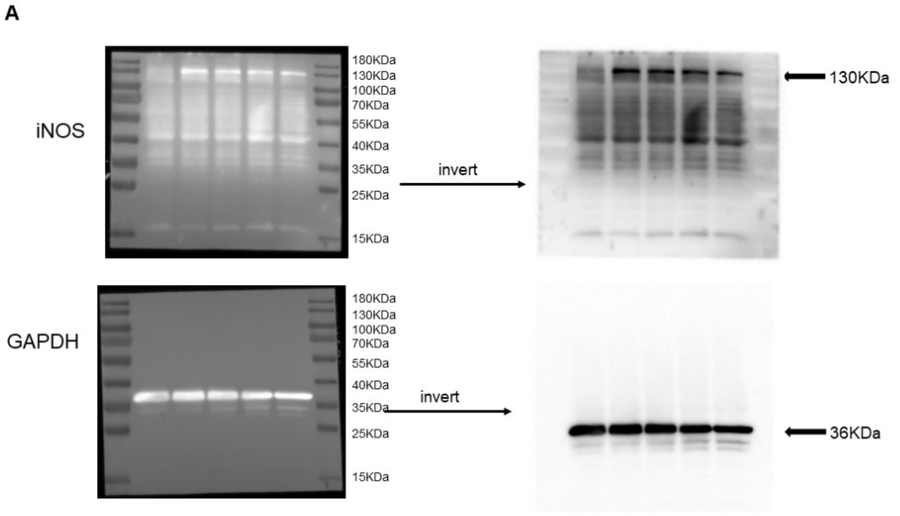

Supplementary Figure 1A iNOS western blot analysis with molecular weight marker

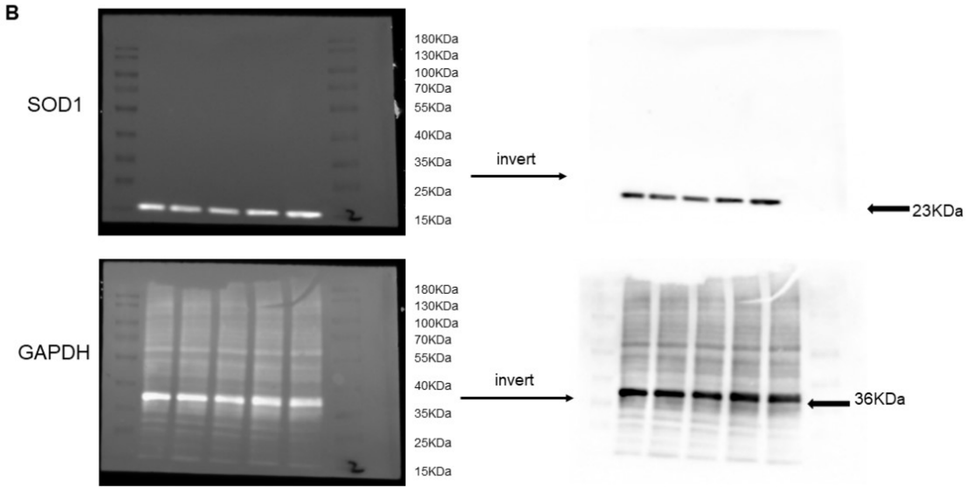

Supplementary Figure 1B SOD1western blot analysis with molecular weight marker

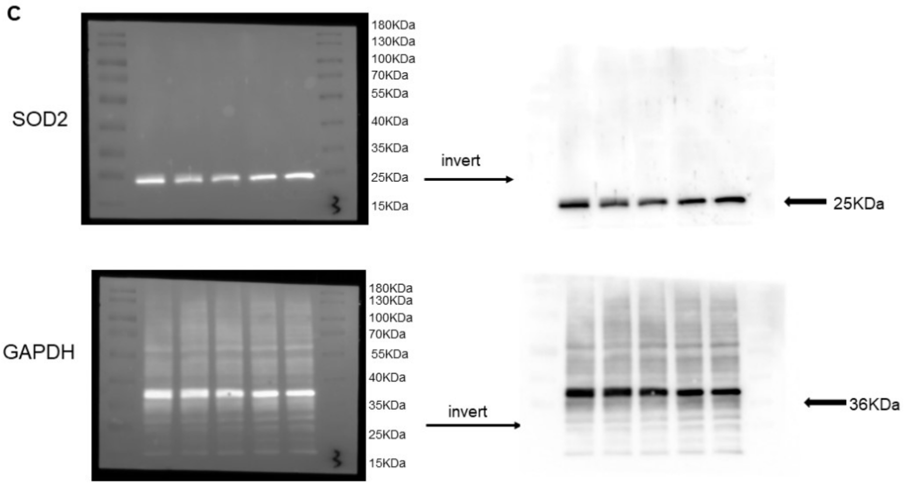

Supplementary Figure 1C SOD2 western blot analysis with molecular weight marker
